# *SOD2* rs4880 and *GPX1* rs1050450 polymorphisms do not confer risk of COVID-19, but influence inflammation or coagulation parameters in Serbian cohort

**DOI:** 10.1080/13510002.2022.2057707

**Published:** 2022-03-31

**Authors:** Djurdja Jerotic, Jovan Ranin, Zoran Bukumiric, Tatjana Djukic, Vesna Coric, Ana Savic-Radojevic, Nevena Todorovic, Milika Asanin, Marko Ercegovac, Ivana Milosevic, Marija Pljesa-Ercegovac, Goran Stevanovic, Marija Matic, Tatjana Simic

**Affiliations:** a Faculty of Medicine, University of Belgrade, Belgrade, Serbia; b Institute of Medical and Clinical Biochemistry, Faculty of Medicine, University of Belgrade, Belgrade, Serbia; c Clinic of Infectious and Tropical Diseases, Clinical Centre of Serbia, Belgrade, Serbia; d Institute of Medical Statistics and Informatics, Faculty of Medicine, University of Belgrade, Belgrade, Serbia; e Clinic of Cardiology, Clinical Centre of Serbia, Belgrade, Serbia; f Clinic of Neurology, Clinical Centre of Serbia, Belgrade, Serbia; g Serbian Academy of Sciences and Arts, Belgrade, Serbia

**Keywords:** COVID-19, polymorphisms, inflammation, thrombosis, GSTP1, SOD2, GPX, Nrf2‌

## Abstract

**Objectives:** Due to the role of oxidative stress in the pathophysiology of COVID-19, it is biologically plausible that inter-individual differences in patients’ clinical manifestations might be affected by antioxidant genetic profile. The aim of our study was to assess the distribution of antioxidant genetic polymorphisms *Nrf2* rs6721961, *SOD2* rs4880, *GPX1* rs1050450, *GPX3* rs8177412, and *GSTP1* (rs1695 and rs1138272) haplotype in COVID-19 patients and controls, with special emphasis on their association with laboratory biochemical parameters.

**Methods:** The antioxidant genetic polymorphisms were assessed by appropriate PCR methods in 229 COVID-19 patients and 229 matched healthy individuals.

**Results:** Among examined polymorphisms, only *GSTP1* haplotype was associated with COVID-19 risk (*p* = 0.009). Polymorphisms of *SOD2* and *GPX1* influenced COVID-19 patients’ laboratory biochemical profile: SOD2*Val allele was associated with increased levels of fibrinogen (*p* = 0.040) and ferritin (*p* = 0.033), whereas GPX1*Leu allele was associated with D-dimmer (*p* = 0.009).

**Discussion:** Our findings regarding the influence of *SOD2* and *GPX1* polymorphisms on inflammation and coagulation parameters might be of clinical importance. If confirmed in larger cohorts, these developments could provide a more personalized approach for better recognition of patients prone to thrombosis and those for the need of targeted antiox­idant therapy.

## 1. Introduction

Oxidative stress (OS) has been recently proposed as a key player in severe acute respiratory syndrome Coronavirus (SARS-CoV-2) infection [1,2]. Several studies have suggested that a prooxidant environment is important for the interaction between viral receptor binding domen and its cellular receptor [1,3–6]. Namely, binding of the SARS-CoV-2 spike S protein to ACE2 receptor of the host cell might depend on conformational changes of these two proteins by the formation of protein disulfide. Indeed, study of Giustarini et al. [6] showed that there is an age-dependent decline of low molecular weight thiol/disulfide ratio of the extracellular fluids, which could play a role in promoting the protein–protein interaction of SARS-CoV-2 and the host cell in the airways. OS is supposed to mediate enhanced cytokine production and cell death [7]. Moreover, major non-communicable diseases, recognized to pose a risk of severe form of COVID-19, are characterized by accumulated oxidative damage [8]. In COVID-19, both highly reactive oxygen and nitrogen species are produced, which target biologically important macromolecules [1,8–10]. Despite relatively well-described biomarkers of oxidative damage in SARS-CoV-2 infection, the data on the role of both regulatory and catalytic antioxidant proteins are scarce and conflicting.

In COVID-19, recent findings pointed to the importance of polymorphisms in glutathione transferase (GST) superfamily of proteins that belong to the first line of enzymatic antioxidant defense. Concisely, COVID-19 patients with *GSTT1-null* genotype exhibit higher mortality and according to our latest results, *GSTP1* (rs1695 and rs1138272) polymorphisms influence susceptibility and severity of COVID-19 [8,11]. Genes encoding both regulatory and catalytic antioxidant proteins exhibit relatively frequent genetic polymorphisms, resulting in complete lack, or altered enzyme activity. These genetic variations result in great inter-individual differences in antioxidant capacity giving each individual a unique and complex antioxidant profile. One of the most studied Nrf2 single nucleotide polymorphisms (SNP) is rs6721961 (-617C/A), which is located in the promoter region. This SNP is found to reduce the transcription activity of Nrf2, associated with attenuated binding of Nrf2 to the ARE, further resulting in decreased Nrf2-dependent gene transcription [12]. Another important SNP is the *SOD2* (rs4880) gene polymorphism which consists of nucleotide substitution (T, thymine → C, cytosine), causing an amino acid substitution of valine (Val) with alanine (Ala). It has been shown that the presence of a variant, SOD2*Val, allele reduces the efficiency of SOD2 transport in mitochondria by 30–40% [13,14]. Regarding *GPX1* (rs1050450) gene polymorphism, it is characterized by nucleotide substitution (C, cytosine → T, thymine), which results in the substitution of the amino acid proline (Pro) with leucine (Leu), leading to decrease in GPX1 activity [15–17]. Polymorphism in gene encoding GPX3 (rs8177412) is a part of GPX3 promoter haplotype responsible for downregulation of gene transcription, resulting in decreased plasma GPX3 activity [18].

Due to the established role of OS in the pathophysiology of COVID-19, it is biologically plausible that inter-individual differences in patients’ clinical manifestations might be affected by antioxidant genetic profile. However, the data on the antioxidant defense system, a crucial determinant of redox balance, are lacking. Therefore, the aim of our study was to assess the distribution of genetic polymorphisms in genes encoding Nrf2, as regulatory, and SOD2, GPX1, GPX3, and GSTP1 haplotype as catalytic antioxidant proteins in COVID-19 patients and respective controls, with special emphasis on their association with laboratory biochemical parameters.

## 2. Material and methods

Study group included 458 Caucasian participants with the Serbian origin. COVID-19 cases were admitted and treated at the clinics for Infectious and Tropical diseases University Clinical Centre of Serbia between July 2020 and February 2021. Patients’ samples were collected between March and June 2021, comprising total of 229 subjects (134 male and 95 female, with an average age of 52.01 ± 12.27 years). Inclusion criteria for the patients were: positive SARS-CoV-2 RT–PCR test performed from nasopharyngeal and oropharyngeal swabs according to World Health Organization guidelines, age ≥ 18 years old and their willingness to provide written informed consent. The control group was matched to COVID-19 patients according to gender and age, and eventually included 229 individuals (124 male, 105 female; average age 50.43 ± 13.16 years). Inclusion criteria for the members of the control group were: negative SARS-CoV-2 RT–PCR according to the available protocols complemented with the absence of detectable SARS-CoV-2 antibodies (IgM and IgG), age ≥ 18 years old and subject's willingness to provide written informed consent. The controls were randomly chosen among subjects exposed to the same infection risks as the patient group in order to obtain the groups of homogeneous origin, and all participants were unrelated.

The Ethical Committee of the Clinical Centre of Serbia (566/01 from 13 July 2020 and 608/01 from 7 August 2020) approved this study and the research was carried out in compliance with the Declaration of Helsinki.

### DNA isolation and genotyping

A total DNA was purified from leukocytes of 200 µl EDTA-anticoagulated blood obtained from the study participants using PureLink™ Genomic DNA Mini Kit (ThermoFisher Scientific, USA).

The *SOD2* rs4880, *GPX3* rs8177412, *GSTP*1(ab) rs1695, and *GSTP1*(cd) rs1138272 polymorphisms were assessed by the real-time PCR, following the manufacturer’s instructions supplied by TaqMan Drug Metabolism Genotyping assays (Life Technologies, Applied Biosystems, USA): C_8709053_10, C_2596717_20, C_3237198_20, and C_1049615_20, respectively. *GPX1* rs1050450 polymorphism was determined by PCR – Restriction Fragment Length Polymorphism (PCR – RFLP) [19], while the *Nrf2* rs6721961 polymorphism was determined by confronting 2-pair primers (CTPP) PCR method [20].

### Statistical analysis

Statistical data analysis was performed using IBM SPSS Statistics 22 (SPSS Inc., Chicago, IL, USA). Results were presented as frequency, per cent, mean ± SD or median (Min–Max). After initial testing for data normality, Student’s *t*-test, Mann–Whitney or Kruskal–Wallis tests were used to compare continuous variables, where appropriate. Differences between categorical variables, as well as Hardy–Weinberg equilibrium for respective genotypes were tested using χ^2^-test. Univariate and multivariate logistic regression was used to assess the association of antioxidant genes polymorphisms and odds for the development of COVID-19. Odds ratio (OR) with 95% confidence interval (CI) was computed after adjusting for the confounding factors: age, gender, presence of hypertension, diabetes mellitus, obesity, and smoking habits. Level of statistical significance was set at *p *< 0.05.

## 3. Results

Selected baseline characteristics of 229 patients, diagnosed with COVID-19 and 229 respective controls are presented in Table 1. As indicated, no statistical difference was found in terms of age, gender distribution, as well as the diagnosis of diabetes between COVID-19 patients and control subjects. However, hypertensive and obese subjects exhibited around 2-fold increased risk for COVID-19 development, while smoking alone was associated with a decreased risk of COVID-19 development.

**Table 1.** Baseline characteristic of COVID-19 patients and respective controls.

Parameters	COVID-19 patients	Controls	OR (95%CI)	*P*
Age (years)^a^	52.01 ± 12.27	50.43 ± 13.16	1.01 (0.99–1.03)	0.186
Gender, n (%)				
Male	134 (58)	124 (54)	1.00^b^	
Female	95 (42)	105 (46)	0.84 (0.58–1.21)	0.346
Hypertension, *n* (%)^c^				
No	82 (54)	142 (70)	1.00^b^	
Yes	71 (46)	62 (30)	1.98 (1.28–3.07)	0.002
Obesity, *n* (%) ^c^				
BMI < 30	149 (67)	158 (83)	1.00^b^	
BMI > 30	75 (33)	32 (17)	2.48 (1.55–3.98)	<0.001
BMI (kg/m^2^)^a^	28.64 ± 5.18	26.15 ± 4.26	1.12 (1.07–1.17)	<0.001
Smoking, *n* (%) ^c^				
Never	114 (52)	85 (38)	1.00^b^	
Former	68 (31)	30 (13)	1.69 (1.01–2.82)	0.045
Ever	36 (17)	111 (49)	0.24 (0.15–0.38)	˂0.001
Diabetes^c^				
No	208 (91)	217 (95)	1.00^b^	
Yes	21 (9)	12 (5)	1.83 (0.88–3.81)	0.108

^a^ Mean ± SD; ^b^Reference group; ^c^Based on available data; CI, confidence interval.

In order to assess the effect of gene polymorphisms on the risk of COVID-19 development, both crude and adjusted OR were calculated (Table 2). Genotypes distribution for all SNPs was in Hardy–Weinberg equilibrium. Among all polymorphisms tested, significant association was found for *GPX3 rs8177412* and *GSTP1 rs1695* and *rs1138272*. Namely, the risk of COVID-19 development was significantly decreased (crude OR = 0.62 95%CI: 0.41–0.95, *p* = 0.027) among carriers of the GPX3*TC + CC genotype compared to the carriers of the GPX3*TT genotype, although significant association was not confirmed by the adjusted analysis (OR = 0.67, 95%CI: 0.38–1.17, *p* = 0.157). In line with our previous findings on smaller cohort, carriers of GSTP1*IleVal + ValVal and GSTP1*AlaVal + ValVal genotype exhibited significantly lower risk of COVID-19 development (crude OR = 0.61, 95%CI: 0.41–0.88, *p* = 0.009 and crude OR = 0.56, 95% CI: 0.37–0.84, *p* = 0.005, respectively) compared to wild-type genotypes, which was even more pronounced in the adjusted analysis (OR = 0.38, 95%CI: 0.22–0.65, *p *˂ 0.001 and OR = 0.42, 95%CI: 0.24–0.73, *p* = 0.002, respectively). Regarding *Nrf2* polymorphism, the obtained results showed the lack of the effect on COVID-19 risk. *SOD2* and *GPX1* genotypes independently did not seem to significantly affect the risk for COVID-19 development as well. The examined polymorphisms of *Nrf2, SOD2, GPX1* and *GPX3* in our study also did not have association with severity of COVID-19 (Table A1, Appendix).

**Table 2.** The distribution of genotypes among COVID-19 patients and controls.

*G*enotype	COVID-19 patients *n*, %	Controls *n*, %	Crude OR (95%CI)^a^	*p*	Adjusted OR (95%CI)^c^	*p*
*SOD2 (rs4880)*						
*Ala/Ala*	58 (25)	63 (28)	1.00^b^		1.00^b^	
*Ala/Val*	118 (52)	121 (53)	1.06 (0.68–1.64)	0.796	0.97 (0.53–1.78)	0.919
*Val/Val*	52 (23)	43 (19)	1.31 (0.77–2.25)	0.321	1.33 (0.64–2.75)	0.441
*Ala/Val + Val/Val*	170 (75)	164 (72)	1.13 (0.74–1.71)	0.576	1.07 (0.59–1.90)	0.829
*GPX1 (rs1050450)*						
*Pro/Pro*	100 (45)	95 (43)	1.00^b^		1.00^b^	
*Pro/Leu*	95 (43)	103 (47)	0.88 (0.59–1.30)	0.513	0.97 (0.57–1.69)	0.898
*Leu/Leu*	27 (12)	22 (10)	1.17 (0.62–2.19)	0.632	1.33 (0.55–3.23)	0.531
*Pro/Leu + Leu/Leu*	122 (55)	125 (57)	0.93 (0.64–1.35)	0.693	1.02 (0.62–1.69)	0.930
*GPX3 (rs8177412)*						
*TT*	173 (76)	142 (67)	1.00^b^		1.00^b^	
*TC*	50 (22)	65 (30)	0.63 (0.41–0.97)	0.036	0.65 (0.36–1.16)	0.145
*CC*	4 (2)	6 (3)	0.55 (0.15–1.98)	0.358	0.87 (0.17–4.39)	0.871
*TC + CC*	54 (24)	71 (33)	0.62 (0.41–0.95)	0.027	0.67 (0.38–1.17)	0.157
*Nrf2 (rs672196)*						
*CC*	159 (71)	163 (77)	1.00^b^		1.00^b^	
*CA*	60 (27)	46 (22)	1.34 (0.86–2.08)	0.197	1.39 (0.75–2.57)	0.294
*AA*	5 (2)	2 (1)	2.56 (0.49–13.40)	0.265	6.67 (0.61–72.89)	0.120
*CA + AA*	65 (29)	48 (23)	1.39 (0.90–2.14)	0.137	1.52 (0.83–2.77)	0.168
*GSTP1 (rs1695)*						
*Ile/Ile*	103 (46)	77 (34)	1.00^b^		1.00b	
*Ile/Val*	102 (45)	125 (54)	0.61 (0.41–0.91)	0.014	0.37 (0.21–0.64)	˂0.001
*Val/Val*	21 (9)	27 (12)	0.58 (0.31–1.11)	0.098	0.45 (0.18–1.11)	0.083
*Ile/Val + Val/Val*	123 (54)	152 (66)	0.61 (0.41–0.88)	0.009	0.38 (0.22–0.65)	˂0.001
*GSTP1* (*rs1138272)*						
*Ala/Ala*	172 (76)	142 (64)	1.00^b^		1.00^b^	
*Ala/Val*	52 (23)	70 (31)	0.61 (0.40–0.94)	0.023	0.45 (0.26–0.79)	0.006
*Val/Val*	2 (1)	10 (5)	0.16 (0.04–0.77)	0.021	0.11 (0.01–1.02)	0.052
*Ala/Val + Val/Val*	54 (24)	80 (36)	0.56 (0.37–0.84)	0.005	0.42 (0.24–0.73)	0.002

^a^ OR, crude odds ratio; CI, confidence interval; ^b^Reference group; ^c^OR, odds ratio adjusted for gender, age, hypertension, diabetes mellitus, smoking, and obesity.

Our analysis on the role of assessed polymorphisms in susceptibility to COVID-19 was further evaluated by performing a haplotype analysis. The linkage disequilibrium (LD) was estimated between *GSTP* polymorphisms by evaluating the nonrandom association of *GSTP* alleles using normalized coefficient of LD (D’). We found a D’ of 0.73 between *GSTP1* (rs1695) and *GSTP1* (rs1138272) (*p* < 0.01), indicative of high association between these pairs of SNPs. As shown in Table 3, the most frequent haplotype among controls (50%) and patients (60%) is H1, taken as a reference group for the analysis. The second most frequent is H2 haplotype was associated with significantly lower risk of COVID-19 development (adjusted OR = 0.43, 95%CI: 0.23–0.81, *p* = 0.009). We found that carriers of H4 haplotype, exhibited the lowest risk of COVID-19 development (OR = 0.36, 95%CI: 0.17–0.74, *p* = 0.006) (Table 3).

**Table 3.** Haplotypes of *GSTP1* (rs1695) and *GSTP1* (rs1138272) in relation to the risk of COVID-19.

Haplotype	*GSTP1 rs1695*	*GSTP1 rs1138272*	Controls %	COVID-19 Patients %	Crude OR (95% CI)^a^	*p*	Adjusted OR (95% CI)^a^	*p*
H1	*A	*C	50	60	1^b^		1^b^	
H2	*G	*C	30	28	0.75 (0.53–1.05)	0.097	0.43 (0.23–0.81)	0.009
H3	*A	*T	11	8	0.65 (0.37–1.14)	0.130	0.44 (0.17–1.12)	0.087
H4	*G	*T	9	4	0.36 (0.17–0.74)	0.006	0.56 (0.15–2.10)	0.39

Global haplotype association *p*-value = 0.0022 for crude analysis and *p*-value = 0.0035 for adjusted analysis; ^a^OR, crude odds ratio; CI, confidence interval; ^b^Reference group; ^c^OR, odds ratio adjusted for gender, age, hypertension, diabetes mellitus, smoking and obesity.

**Figure 1.**
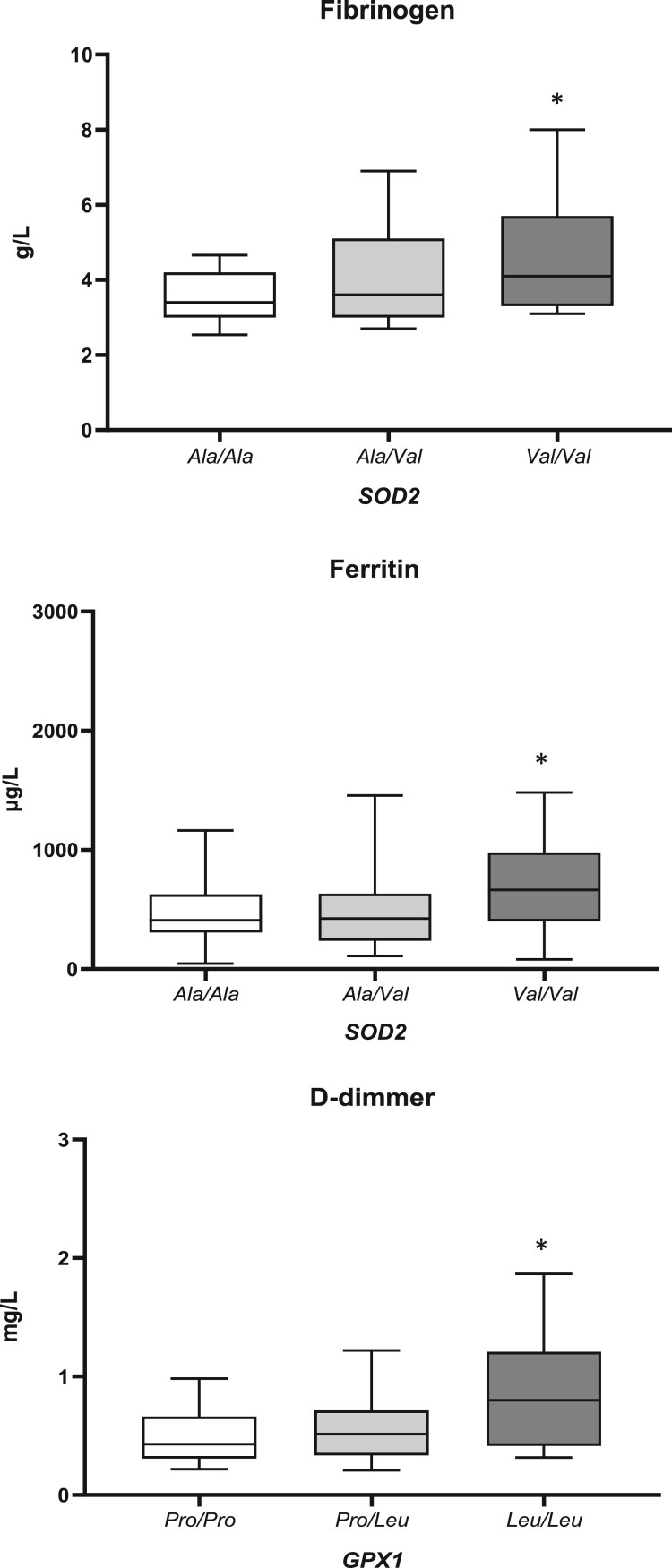
The associations between *SOD2* and *GPX1* polymorphisms and levels of ferritin, fibrinogen, and D-dimmer. Results are presented as the median with interquartile range; *p < 0.05.

Inflammation and coagulation parameters, obtained from the COVID-19 patients upon admission to the Hospital, with regard to assessed genotypes are presented in Table 4. COVID-19 patients homozygous for variant SOD2*Val/Val genotype, had increased levels of both fibrinogen (*p* = 0.040) and ferritin (*p* = 0.033) (Figure 1). Moreover, the presence of GPX1*Leu/Leu genotype was found to be significantly associated with increased levels of D-dimmer (*p* = 0.009) (Figure 1). COVID-19 patients homozygous for variant GPX1*Leu/Leu had the highest levels of fibrinogen as well, but the significance was borderline (*p* = 0.089). Regarding other inflammatory parameters, we observed similar trend for CRP and IL-6, but still without reaching statistical significance.

**Table 4.** The association between antioxidant gene polymorphisms and levels of CRP, IL-6, Ferritin, Fibrinogen, and D-dimmer.

Genotype	CRP (mg/L)^a^	*p*	IL-6 (pg/mL)^a^	*p*	Ferritin (µg/L)^a^	*p*	Fibrinogen (g/L)^a^	*P*	D-dimmer (mg/L FEU) ^a^	*p*
*SOD2 (rs4880)*										
*Ala/Ala*	23.75 (0.6–164.4)		19.5 (1.5–133.9)		409 (29.2–1446.9)		3.4 (2.2–5.6)		0.55 (0.19–10.8)	
*Ala/Val*	30.8 (0.5–282.2)		22.25 (1.5–213)		424.2 (10.1–4579.2)		3.6 (2.1–8)		0.45 (0.18–8.26)	
*Val/Val*	43.4 (1.1–280.5)	0.352	27.6 (1.4–196.9)	0.521	664.2 (36.7–4937.2)	0.033	4.1 (2.3–9)	0.040	0.49 (0.19–2.15)	0.635
*GPX1 (rs1050450)*										
*Pro/Pro*	26 (0.6–188.9)		21.6 (1.5–168.8)		454.7 (10.1–2001.1)		3.4 (2.2–8)		0.43 (0.18–10.8)	
*Pro/Leu*	31.25 (0.5–282.2)		23.35 (1.4–213)		451.95 (29.2–4937.2)		3.6 (2.1–9)		0.52 (0.19–8.26)	
*Leu/Leu*	58.6 (1–224.5)	0.354	35.7(1.8–205.5)	0.484	576.4 (13.6–4579)	0.815	4.45 (2.7–8)	0.083	0.8 (0.31–2.7)	0.009
*GPX3* (*rs8177412)*										
*TT*	33.75 (0.5–280.5)		23.75 (1.4–213)		470.75 (10.1–4579.2)		3.65 (2.1–8)		0.5 (0.18–10.8)	
*TC*	17.5 (1.1–282.2)		16 (1.5–168.2)		454.7 (13.6–4937.2)		3.45 (2.3–9)		0.41 (0.19–1.44)	
*CC*	14.75 (1.6–27.9)	0.285	19.9 (10.6–29.2)	0.386	147.95 (59–236.2)	0.130	2.9 (2.9–2.9)	0.363	0.31 (0.29–0.33)	0.106
*Nrf2 (rs672196)*										
*CC*	33.75 (0.5–282.2)		23.8 (1.4–213)		479.6 (10.1–4937.2)		3.8 (2.2–9)		0.49 (0.18–10.8)	
*CA*	18.6 (0.6–224.5)		18 (1.5–133.9)		455.4 (29.2–2840.5)		3.4 (2.1–8)		0.45 (0.19–1.44)	
*AA*	16.45 (1–31.9)	0.178	/	0.310	/	0.771	3.5 (3.4–3.6)	0.138	0.7 (0.61–0.8)	0.571
*GSTP1 (rs1695)*										
*Ile/Ile*	28.8 (0.6–282.2)		21.2 (1.4–168.2)		411.1 (10.1–4937.2)		3.6 (2.4–8)		0.49 (0.19–8.26)	
*Ile/Val*	31.9 (0.5–280.5)		22.6 (1.5–213)		461.9 (13.6–3037.9)		3.85 (2.1–9)		0.46 (0.18–10.8)	
*Val/Val*	29 (1.6–164.7)	0.711	32.8 (1.8–115.1)	0.460	581.4 (36.7–2840.5)	0.216	3.35 (2.2–8)	0.370	0.39 (0.22–1.23)	0.513
*GSTP1* (*rs1138272)*										
*Ala/Ala*	28.45 (0.5–282.2)		19.65 (1.4–196.9)		437.5 (10.1–4937.2)		3.6 (2.1–8)		0.46 (0.18–10.8)	
*Ala/Val*	42.7 (1–158.8)		33.9 (1.5–213)		551.3 (42–1446.9)		3.65 (2.3–9)		0.5 (0.19–8.26)	
*Val/Val*	5 (5–5)	0.629	/	0.052	/	0.511	/	0.629	/	0.449

^a^ Median (Min–Max).

## 4. Discussion

Since OS plays an important role in SARS-CoV-2 infection, we speculated that variations in catalytic and regulatory antioxidant proteins modulate susceptibility towards COVID-19. The data obtained herewith have shown that among examined polymorphisms, only *GSTP1* haplotype (rs1695 and rs1138272) was associated with COVID-19 risk. Additionally, we have found that polymorphisms of *SOD2* rs4880 and *GPX1* rs1050450 influence COVID-19 patients’ laboratory biochemical profile. Namely, SOD2*Val allele was significantly associated with increased levels of both fibrinogen and ferritin, whereas GPX1*Leu allele was associated with higher levels of fibrinogen, and especially D-dimmer.

Among the first results in the field of antioxidant protection in COVID-19 were data on reduced erythrocyte activity of glutathione peroxidase, catalase, and superoxide dismutase in these patients compared to healthy individuals [21]. We hypothesized that variant *GPX1* allele would potentiate this reduction in activity, since purified GPX1*Leu variant has been shown to possess lower enzymatic activity compared to the GPX1*Pro enzyme [16]. Besides, particular importance of GPX1 in COVID-19 infection was suggested by the findings that GPX1 can function as a binding partner for main protease of SARS-CoV-2 virus (Mpro) [22]. This was supposed to result in Mpro inhibition, denoting GPX1 and MproSARS-CoV-2 interaction as a new therapeutic molecular target. However, our results on the lack of association of *GPX1* polymorphism with susceptibility to COVID-19 are not in favor of the presumption that GPX1 acts as a Mpro inhibitor. On the other hand, it seems that polymorphic GPX1 expression influences coagulation, since we found that COVID-19 patients with low-activity GPX1*Leu allele had higher levels of both fibrinogen and D-dimmer. SARS-CoV-2 infection is accompanied by hypercoagulability, which leads to high morbidity and mortality of patients with COVID-19 [23,24]. There are several mechanisms by which GPX1 might protect against accelerated thrombosis. Concisely, overexpression of GPX1 in aged mice protects against the accelerated thrombosis, via decreasing platelet hyperresponsiveness mediated by H_2_O_2_ [25]. Moreover, the reduction of OS by GPX1, protects against post-translational modifications of fibrinogen exerted by ROS and NO-derived oxidants that increase its thrombogenicity [26,27]. Additionally, owing to the antioxidant actions of GPX1 in removing intracellular hydrogen peroxide, GPX1 plays an essential role in preserving endothelial function and NO bioavailability [28]. This was extrapolated by the findings on prospective human study reporting that increasing erythrocyte GPX1 activity reduces the risk of cardiovascular events [29]. Conversely, low-activity GPX1*Leu allele was found to be associated with in-stent restenosis, which confirms the significance of this polymorphism in accelerated thrombosis [30]. Thus, therapeutic strategies lowering platelet H_2_O_2_ levels, including GPX1 mimetics such as ebselen, may have the potential to decrease thrombotic complications [25]. Larger studies are needed to confirm whether *GPX1* polymorphism can serve as a biomarker of increased susceptibility to enhanced coagulation in COVID-19.

We also analyzed polymorphic expression of GPX3, which is the only extracellular member of the GPX family. Although initial results obtained in our study have shown that the presence of at least one GPX3*C allele reduces the risk of COVID-19, such significant effect was not achieved when adjusted analysis was performed. Similarly, *SOD2* polymorphism (rs4880) had no effect on the susceptibility to COVID-19. However, we found significant association between SOD2*Val allele and increased levels of inflammatory parameters, such as fibrinogen and ferritin. Regarding other inflammatory parameters, we observed similar trend for CRP and IL-6, but still without reaching statistical significance. The SOD2*Val allele reduces the efficiency of SOD2 transport in mitochondria by 30–40%, thus resulting in lower dismutation of superoxide anion into H_2_O_2_ [13,14]. It has been shown that the accumulation of superoxide anion, in the individuals carrying SOD2*Val/Val genotype, have a pro-inflammatory role [31]. Moreover, recent studies have demonstrated that SOD2 may be potential anti-inflammatory agent due to its ability to scavenge superoxide anion [31,32]. Indeed, the results of Montano et al. [32] showed that *SOD2* rs4880 polymorphism influences inflammatory immune response. Namely, they reported that SOD2*Val/Val human peripheral blood mononuclear cells (PBMCs) have higher levels of pro-inflammatory cytokines IL-1, IL-6, TNF-a, IFN-γ, when compared to SOD2*Ala/Ala PBMCs. Our data aligns with these findings, since we found that COVID-19 patients with less efficient SOD2*Val variant presented higher susceptibility to the inflammatory process. Moreover, there are several types of SOD2 mimetics that have a positive effect on the inflammatory response of lung epithelial cells in preclinical models of chronic obstructive pulmonary disease [33]. The use of the contrast agent Mangafodipir, which acts as SOD2 mimetic, has shown favorable anti-inflammatory results in clinical trials [34]. The second phase of clinical trials of the effects of SOD2 mimetics in patients with COVID-19 is underway.

As mentioned before, our latest findings showed that both genetic variants in *GSTP1*, *Ile105Val* (rs1695), and *Ala114Val* (rs1138272) are associated with susceptibility and severity of COVID-19 [11]. Herein, we further substantiated results by haplotype analysis of *GSTP1* genotypes and found that carriers of H2 haplotype (presence of *GSTP1* rs1695 variant allele and *GSTP1* rs1138272 referent allele) exhibited the lowest risk of susceptibility of COVID-19. In view of the fact that GSTP1 is highly expressed in lung tissue and might even be considered as predominant GST in the lungs, this result is biologically plausible [11,35–38]. As previously discussed, GSTP1 might modulate the susceptibility to various pulmonary diseases [11]. The question arrases why examined polymorphisms of immediate and first line antioxidant enzymes SOD2, GPX1, and GPX3 in our study did not have association either with susceptibility or severity of COVID-19, despite the fact that redox homeostasis misbalance is one of the significant underlying mechanisms in COVID-19 disease. We speculate that cytokine and free radical storm, indubitably shown in COVID-19 patients, overwhelm the antioxidant protection given by the presence of referent genotypes of the immediate and first line antioxidant enzymes. Since GSTP1 is predominantly expressed in the lungs, this might be the reason why it influenced the susceptibility and severity of COVID-19 disease.

In summary, our results on the association between antioxidant genetic profile and severity of clinical manifestations in COVID-19 patients may contribute to further understanding of pathophysiological mechanisms underlining this disease. Our findings regarding the influence of *SOD2* and *GPX1* polymorphisms on the laboratory biochemical parameters might be of clinical importance, since we found that COVID-19 patients with low-activity alleles of these genes have higher levels of inflammation and coagulation parameters. New developments in the field of antioxidant polymorphisms in COVID-19 patients could provide a more personalized approach for better recognition of patients prone to thrombosis and for the need of targeted antioxidant therapy.

## Supplementary Material

Supplemental Material

## Data Availability

The data supporting reported results are available at *RedCap* platform *(Research Electronic Data Capture, Vanderbilt University)* of Faculty of Medicine University in Belgrade and will be made available by the corresponding authors upon request without undue reservation.
