# Supplementary material for: SOD2 rs4880 and GPX1 rs1050450 polymorphisms do not confer risk of COVID-19, but influence inflammation or coagulation parameters in Serbian cohort
Source: Redox Rep. 2022 Mar 31;27(1):85–91. doi: 10.1080/13510002.2022.2057707 (PMC8979533; doi:10.1080/13510002.2022.2057707)
Supplement: Supplemental Material [file YRER_A_2057707_SM4694.doc]

**Table A1. The association of *SOD2, GPX1, GPX3* and *Nrf2* polymorphisms with severity of COVID-19**

| ***G*enotype** | **Mild COVID-19**  **n, %** | **Severe COVID-19**  **n, %** | **Crude**  **OR (95%CI)**^a^ | ***p*** | **Adjusted**  **OR (95%CI)**^c^ | ***p*** |
| --- | --- | --- | --- | --- | --- | --- |
| ***SOD2 (rs4880)*** |  |  |  |  |  |  |
| *Ala/Ala* | 22 (28) | 35 (24) | 1.00^b^ |  | 1.00^b^ |  |
| *Ala/Val* | 39 (50) | 77 (53) | 1.24 (0.64-2.39) | 0.520 | 1.09 (0.39-3.02) | 0.875 |
| *Val/Val* | 17 (22) | 34 (23) | 1.26 (0.57-2.77) | 0.570 | 0.99 (0.31-3.20) | 0.983 |
| ***GPX1 (rs1050450)*** |  |  |  |  |  |  |
| *Pro/Pro* | 32 (42) | 65 (46) | 1.00^b^ |  | 1.00^b^ |  |
| *Pro/Leu* | 34 (44) | 60 (43) | 0.87 (0.48-1.58) | 0.644 | 0.55 (0.24-1.41) | 0.212 |
| *Leu/Leu* | 11 (14) | 16 (11) | 0.72 (0.30-1.72) | 0.455 | 0.62 (0.15-2.65) | 0.518 |
| ***GPX3 (rs8177412)*** |  |  |  |  |  |  |
| *TT* | 57 (72) | 112 (78) | 1.00^b^ |  | 1.00^b^ |  |
| *TC* | 19 (24) | 31 (22) | 0.83 (0.43-1.60) | 0.557 | 0.78 (0.28-2.18) | 0.635 |
| *CC* | 3 (4) | 1 (1) | 0.17 (0.02-1.70) | 0.128 | 0.11 (0.01-3.02) | 0.193 |
| ***Nrf2 (rs672196)*** |  |  |  |  |  |  |
| *CC* | 49 (63) | 107 (75) | 1.00^b^ |  | 1.00^b^ |  |
| *CA* | 26 (33) | 34 (24) | 0.60 (0.33-1.11) | 0.101 | 0.57 (0.20-1.60) | 0.284 |
| *AA* | 3 (4) | 1 (1) | 0.15 (0.02-1.51) | 0.107 | 0.36 (0.01-13.32) | 0.580 |

Mild COVID-19: Stage I; Severe COVID-19: Stages II+III+IV;  ^a^OR, crude odds ratio; CI, confidence interval; ^b^Reference group ; ^c^OR, odds ratio adjusted for gender, age, hypertension, diabetes mellitus, smoking and obesity
